# Strobilurin X acts as an anticancer drug by inhibiting protein synthesis and suppressing mitochondrial respiratory chain activity

**DOI:** 10.1007/s12672-024-01041-w

**Published:** 2024-05-20

**Authors:** Kenji Takahashi, Tomoya Tanaka, Atsushi Ishihara, Toshio Ohta

**Affiliations:** 1 Department of Veterinary Pharmacology, Faculty of Agriculture, Tottori University, Tottori, 680-8553 Japan; 2 Division of Functional Fungal Physiology and Pharmacology, Fungus/Mushroom Resource and Research Center, Faculty of Agriculture, Tottori University, Tottori, 680-8553 Japan; 3 Graduate School of Sustainability Sciences, Tottori University, Tottori, 680-8553 Japan; 4 Division of Applied Fungal Chemistry, Fungus/Mushroom Resource and Research Center, Faculty of Agriculture, Tottori University, Tottori, 680-8553 Japan

**Keywords:** *Mucidula venosolamellata*, Strobilurin X, Anticancer, Mitochondrial respiratory chain complex III, Protein synthesis

## Abstract

**Purpose:**

Strobilurins act as antifungal agents by inhibiting the mitochondrial respiratory chain. The cytotoxic activity of strobilurins, focusing on its anticancer activities, has been reported. However, the mechanisms involved in these activities remain unclear.

**Methods:**

The cytotoxic effects of strobilurin X isolated from the mycelium of *Mucidula. venosolamellata* were examined in human cancer cell lines (A549 and HeLa) and normal fibroblasts (WI-38).

**Results:**

Strobilurin X significantly decreased the viability of A549 and HeLa cells compared to that in the WI-38 cells after 48 h of exposure. The EC_50_ values for cytotoxicity in the A549, HeLa, and WI-38 cells were 3.4, 5.4, and 16.8 μg/mL, respectively. Strobilurin X inhibited the mitochondrial respiratory chain and enhanced the release of lactate in the A549 cells. The IC_50_ value of strobilurin X against the mitochondrial respiratory chain complex III activity was 139.8 ng/mL. The cytotoxicity induced by strobilurin X was not completely rescued after adding uridine, methyl pyruvate, or *N*-acetyl cysteine. Furthermore, pharmacological approaches demonstrated that strobilurin X failed to modulate the mitogen-activated protein kinase family and phosphoinositide 3-kinase-Akt pathways; alternatively, it suppressed protein synthesis independent of uridine.

**Conclusion:**

Strobilurin X induced cytotoxicity by blocking the mitochondrial respiratory chain and suppressing protein synthesis. These findings may aid in the development of novel anticancer drugs using strobilurins.

## Introduction

Strobilurins are antifungal pesticides widely used in agriculture across the world [1, 2]. Strobilurins A and B were initially isolated from the mycelium of the basidiomycete *Strobilurus tenacellus* [3]. Mucidin extracted from *Oudemansiella mucida* [4] was structurally identical to strobilurin A [5]. Strobilurins exert antifungal effects by inhibiting the transfer of an electron in the mitochondrial respiratory chain at complex III [5–9]. Several types of strobilurins have been isolated and identified from various mushrooms and synthesized for the development of pesticides [1, 10]. Strobilurin X was detected in the culture medium of *Oudemansiella mucida* and showed the higher antifugal activity than strobilurin A [11, 12].

Cancer is the most common morbidity, leading to a growing demand for the development of novel drugs. Despite many studies on the anticancer effects of various mushroom extracts, the findings have not been exploited for clinical use [13]. The mitochondrial respiratory chain is one of many therapeutic targets for cancer treatments because it is essential for cell proliferation [14]. In one study, the deletion of complex III, one of the mitochondrial respiratory chain components, abolished tumor cell growth, resulting in the amplification of the tumor engrafted in mice [15]. However, no compounds inhibiting complex III have been successfully used to develop anticancer drugs. Strobilurins inhibit the activity of complex III by suppressing cytochrome bc1 [5, 13]; thus, they may be useful for the development of compounds that can inhibit cancer cell proliferation.

The aim of this study was to examine the cytotoxic effects of strobilurin X, isolated from *Mucidula venosolamellata,* on cancer cells. Strobilurin X significantly suppressed cell proliferation and inhibited the mitochondrial respiratory chain complex III activity. However, the cytotoxicity was not solely attributed to the suppression of complex III, indicating that strobilurin X had alternative targets for its cytotoxic effect. Further experiments revealed that strobilurin X inhibited protein synthesis. These results suggested that strobilurin X exerts anticancer effects and may serve as a lead compound for developing new anticancer drugs.

## Methods

### Preparation of strobilurin X

Strobilurin X was extracted from *M. venosolamellata* (TUFC 30333, Fungus/Mushroom Resource and Research Center, Faculty of Agriculture, Tottori University). *M. venosolamellata* was cultured in 5 L of malt extract media for 30–60 days. The culture filtrate was extracted using ethyl acetate (1 L × 3) and fractionated by silica gel column chromatography (acetone-hexane, 10% stepwise from 0 to 30% acetone). The 10% acetone fraction was further fractionated by silica gel column chromatography (ethyl acetate-hexane, 10% stepwise elution from 0 to 30%). Strobilurin X was purified from the 20% ethyl acetate fraction via preparative high-performance liquid chromatography using a Cosmosil 5C18 ARII (10 × 250 mm, Nacalai Tesque, Kyoto, Japan) column and 65% acetonitrile–water as solvent (flow rate: 3 mL/min, ultraviolet detection: 254 nm, [Shimadzu 10A system, Kyoto, Japan]). The identity of the compound was confirmed by nuclear magnetic resonance and mass spectra according to our recent report [16].

### Cell culture

Lung and cervical cancer cell lines (A549 and HeLa, respectively) and normal lung fibroblasts (WI-38) were purchased from Riken Bioresource Center (Tsukuba, Japan) and cultured in Dulbecco’s modified Eagle medium (D6429; Sigma-Aldrich, Tokyo, Japan) supplemented with 10% bovine fatal serum, 100 U/mL streptomycin (Meiji Seika Co. Ltd, Tokyo, Japan) and 100 μg/mL penicillin (Meiji Seika Co. Ltd).

### Detection of cell viability using the WST-8 assay

Cell viability was assessed using Cell Counting Kit-8 (WST-8; Dojin, Tokyo, Japan). In brief, the cells (5 × 10^3^ cells/well) were prepared in a 96-well culture plate the day before the experiment. Various concentrations of strobilurin X were applied for 48 h, and the cell viability was evaluated by measuring the absorbance with a microplate spectrometer (Sunrise^™^ Fuji film Wako Pure Chemicals, Osaka, Japan) at 450 nm between 1 and 4 h after the addition of the WST-8 reagent to the culture medium in each well. The ratio of each data set to that of the vehicle was calculated.

### Quantitation of lactate production from the cells

The amount of lactate produced through glycolysis was measured using the Lactate Assay Kit-WST (Dojin). The cells (10^4^ cells/well) were prepared in the 96-well culture plate the day before the experiment and incubated with various concentrations of strobilurin X for 48 h. A portion of each conditioned medium was transferred to a new 96-well culture plate and mixed with a working solution, according to the operating instructions. After incubation for 30 min at 37 °C, the absorbance of each sample was measured at 450 nm. The amount of lactate produced by the cells under each condition was calculated using a standard curve.

### Measurement of the mitochondrial respiratory chain complex III activity in cell-free system

The mitochondrial respiratory chain complex III activity was measured using the MitoCheck^®^ Complex II/III Activity Assay Kit (Cayman Chemical, Ann Arbor, MI, USA) according to recent reports [17, 18]. In brief, the reduction of cytochrome c by complex III was detected at 550 nm using a spectrophotometer (Genesys 10S UV–Vis; ThermoFisher Scientific, Tokyo, Japan). A working solution of strobilurin X was mixed with cytochrome c solution and bovine heart mitochondria, and the absorbance was measured every 30 s for 10 min. The activity of complex III was calculated as the ratio of the rate of change in absorbance in each sample to that in the vehicle.

### Detection of the protein synthesis activity

The protein synthesis activity was assessed using the Cayman’s Protein Synthesis Assay Kit (Cayman Chemical). In brief, the cells (3 × 10^3^ cells/well) were prepared in a 96-well culture plate with a black bottom (Thermo Fisher Scientific) the day before the experiment. The cells were pretreated with strobilurin X for 30 min and labeled with *O*-Propargyl-puromycin (OPP) for 2 h. The amount of 5-carboxyfluorescein (FAM) bound to OPP was determined by measuring the fluorescence at 535 nm with excitation at 488 nm using a fluorescent microscope (BZ-X810, Keyence, Osaka, Japan).

### Measurement of intracellular reactive oxygen species (ROS) production

After exposure to strobilurin X, the cells harvested by trypsinization were washed with phosphate-buffered saline (PBS) and loaded with chloromethyl-2ʹ,7ʹ—dichlorodihydrofluorescein diacetate (CM-H_2_DCFDA; 40 μM; C6827, Thermo Fisher Scientific) for 30 min at 37 °C. Subsequently, the cells were washed with PBS and analyzed using a flow cytometer (FACS Aria, BD Japan, Tokyo, Japan).

### Chemicals

Uridine (solvent, stock concentration; PBS, 200 mg/ml), methyl pyruvate (PBS, 110 mg/mL) and *N*-acetyl cysteine (1 M NaOH, 1 M) are purchased from FUJIFILM Wako Pure Chemical Corp. (Osaka, Japan). PD98059 (dimethylsulfoxide [DMSO], 50 mM), U0126 (DMSO, 20 mM), LY2940001 (DMSO 5 mM), JNK inhibitor II (DMSO, 10 mM) and SB203580 (DMSO, 10 mM) were obtained from Calbiochem (Sigma-Aldrich Japan, Tokyo, Japan).

### Statistical analysis

All analyses were conducted using the SPSS Statistics software (IBM Japan, Tokyo, Japan). Student’s or Welch’s *t*-test was used for two-group comparisons, and one-way analysis of variance followed by the Games-Hawell test was used for multiple-group comparisons. A p-value of < 0.05 was considered significant.

## Results

### Cytotoxicity induced by strobilurin X

**Fig. 1:**
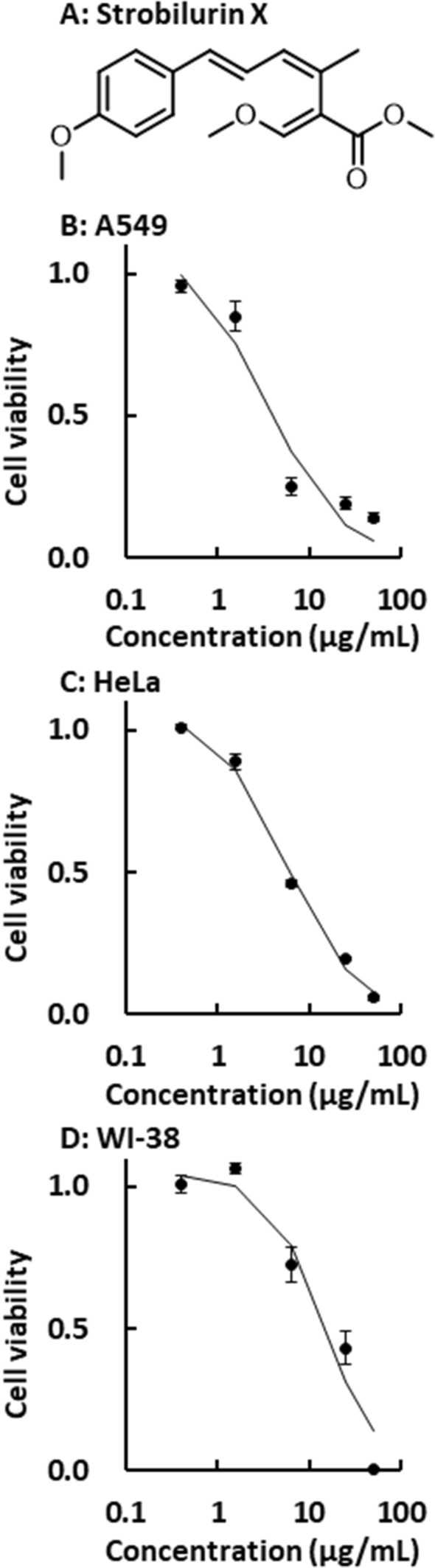
Cytotoxicity induced by strobilurin X. **A** Chemical structure of strobilurin X. Relationships between viability and the concentrations of strobilurin X at 48 h in the A549 **B**, HeLa **C**, and WI-38 **D** cells. Cell viability is indicated as the ratio of each value to that of the vehicle. Symbols with vertical lines show the mean ± the standard error of the mean (SEM) in three separate experiments

Strobilurins have been reported to induce cell death [19, 20]. Therefore, the cytotoxic effect of strobilurin X was examined in the current study. Strobilurin X significantly decreased the viability of the A549 and HeLa cells at 48 h in a concentration-dependent manner. The EC_50_ values of strobilurin X in the A549 and HeLa cells were 3.4 ± 0.2 and 5.4 ± 0.3 μg/mL, respectively (Figs. 1A and B). Although a cytotoxic effect was observed in the WI-38 cells, the EC_50_ value in these cells (16.8 ± 3.4 μg/mL) was higher than those in the two cancer cell lines (Fig. 1C). These results suggest that strobilurin X has a relatively selective cytotoxic effect on cancer cells.

### Involvement of blockade of mitochondrial respiratory chain complex III in strobilurin X-induced cytotoxicity

**Fig. 2:**
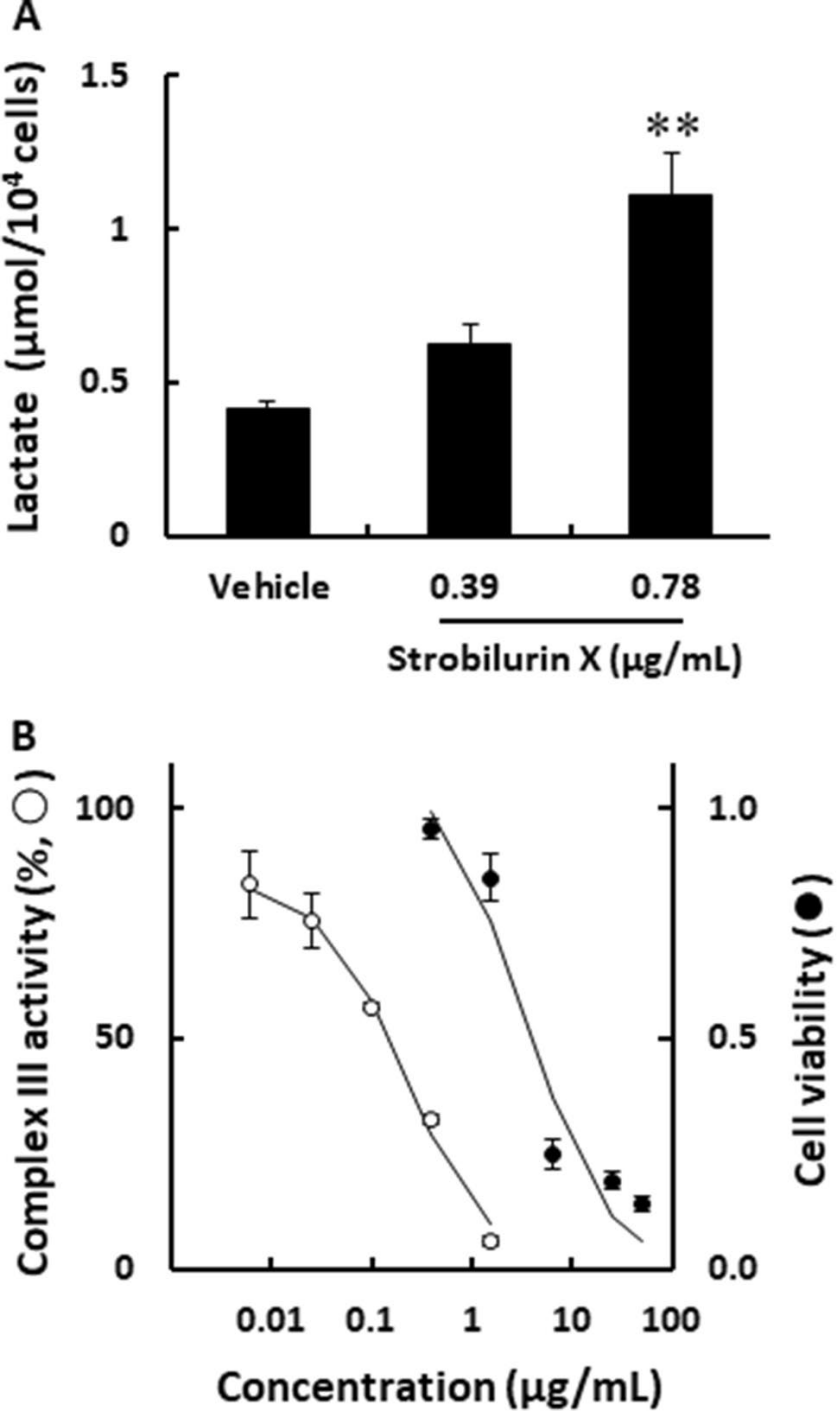
The effects of strobilurin X on mitochondrial respiratory chain activity. **A** The amount of extracellular lactate from A549 cells after stimulation with strobilurin X for 48 h. The column with a vertical line shows the mean ± SEM in three separate experiments. **B** The relationship between the mitochondrial respiratory chain complex III activity (○) or cell viability (●) and the concentrations of strobilurin X. Complex III activity is indicated as the percentage of the rate of change in each activity to that of the vehicle. The cell viability data is transcribed from Fig. 1A. Symbols with vertical lines show the mean ± SEM in three experiments. **p < 0.01 vs. vehicle using the Games-Hawell test

A549 cells were used to evaluate the detail cytotoxic effects and mechanisms of strobilurin X. Inhibition of the mitochondrial respiratory chain enhances the glycolytic system [21]; therefore, the amount of lactate effluxed by glycolysis from the A549 cells was therefore measured in the current study. Strobilurin X increased lactate release at 48 h (Fig. 2A) and inhibited the activity of the mitochondrial respiratory chain complex III in a concentration-dependent manner. The IC_50_ of strobilurin X was 139.8 ± 32.0 ng/mL; however, this value was significantly lower than the concentration showing cytotoxicity in A549 (Fig. 2B). These results clearly showed that strobilurin X inhibited mitochondrial respiratory chain complex III, although the experimental setting was different between the measurement of complex III activity and cytotoxicity. Future experiments using extracts from used cells may reveal details.

**Fig. 3:**
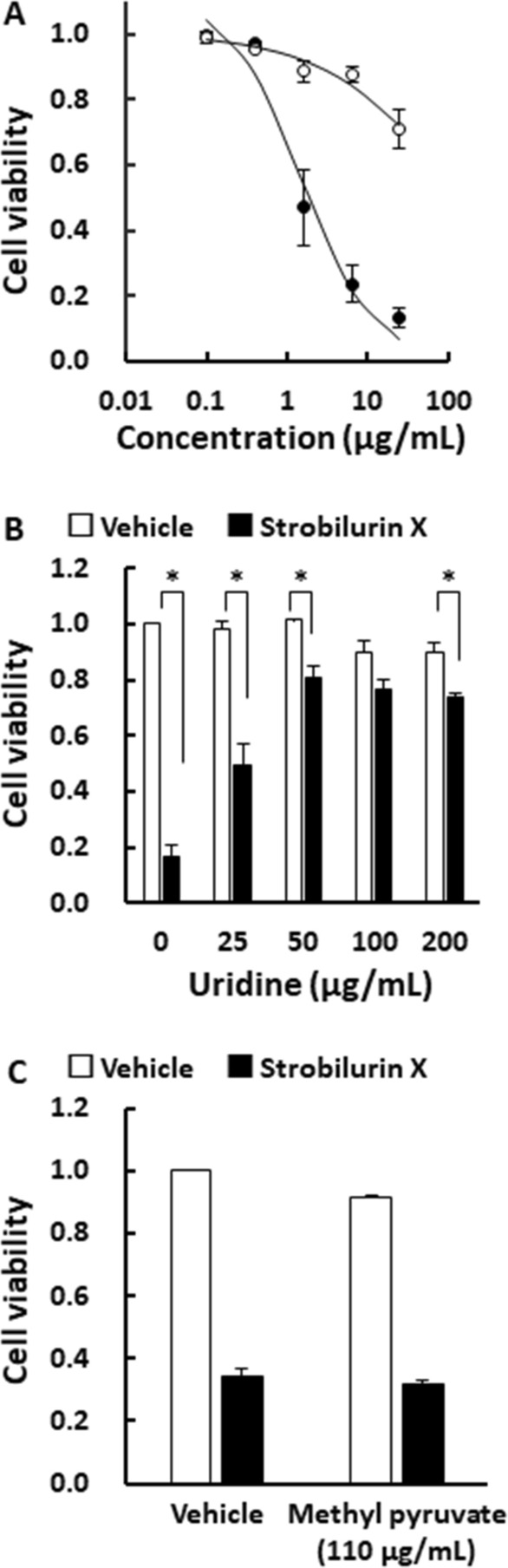
Effects of uridine on the strobilurin X-induced cytotoxicity in A549 cells. **A** Relationships between the cell viability and concentrations of strobilurin X at 48 h without (●) and with uridine (○; 50 μg/mL). Cell viability is indicated as the ratio of each value to that of the vehicle without uridine. Symbols with vertical lines show the mean ± SEM in three separate experiments. **B**, **C** Relationship between the cell viability induced by the vehicle (open columns) and strobilurin X (closed columns, 6.25 μg/mL) and the concentrations of uridine **B** or methyl pyruvate (C; 110 μg/mL) at 48 h. Columns with vertical lines show the mean ± SEM in three separate experiments. *p < 0.05 vs. the vehicle with uridine using Student’s *t*-test or Welch’s *t*-test

As shown in Fig. 3A, uridine (50 μg/mL) significantly reduced the cytotoxicity of strobilurin X, thus indicating that pyrimidine biosynthesis was inhibited by strobilurin X through the mitochondrial respiratory chain. Uridine rescued the cytotoxicity of strobilurin X (6.25 μg/mL) in a concentration-dependent manner, but did not completely overcome the cytotoxic effect even at 200 μg/mL (Fig. 3B). Pyruvate is also known to reduce the cytotoxicity induced by mitochondrial respiratory chain dysfunction [15, 22]. As shown in Fig. 3C, methyl pyruvate did not attenuate the strobilurin X-induced cytotoxic effect. These results suggest that other targets, besides the inhibition of the mitochondrial respiratory chain complex III, may be involved in the cytotoxic action of strobilurin X.

### Inhibition of protein synthesis by strobilurin X

**Fig. 4:**
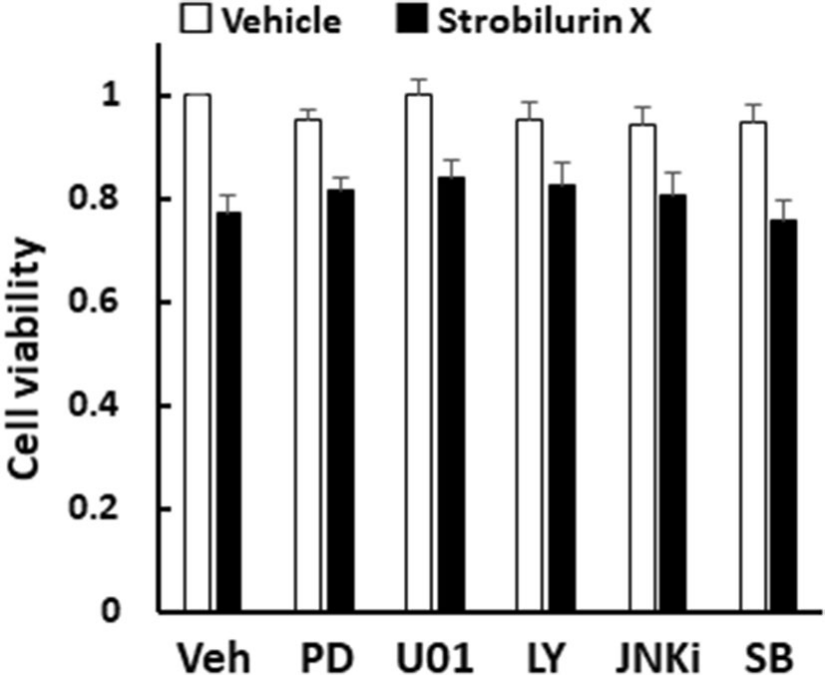
Effects of inhibitors of cell proliferation and death signals on the cytotoxicity induced by strobilurin X in the A549 cells. Cell viability is indicated as the ratio of each value to that of the vehicle without inhibitors at 24 h (open columns, vehicle; closed columns, strobilurin X [6.25 μg/mL]). Veh, vehicle; PD, 10 μM PD98059; U01, 10 μM U0126; LY, 5 μM LY2940004; JNKi, 5 μM JNK inhibitor; SB, 10 μM SB203950. Columns with vertical lines show the mean ± SEM in three separate experiments

Among the cell growth signals, PD98059, U0126, mitogen activated protein kinase (MAPK)/extracellular signal-regulated kinase (Erk) kinase (MEK) inhibitors, and LY2940001, a phosphoinositide 3-kinase (PI3K) inhibitor, failed to modulate the strobilurin X (6.25 μg/mL)-induced cytotoxicity. Likewise, among the cell death signals, JNK inhibitor II and SB203580, a p38 MAPK inhibitor, did not affect the strobilurin X-induced cell death (Fig. 4). These results suggest that the cell growth and cell death signals are not related to the cytotoxic effect of strobilurin X.

**Fig. 5:**
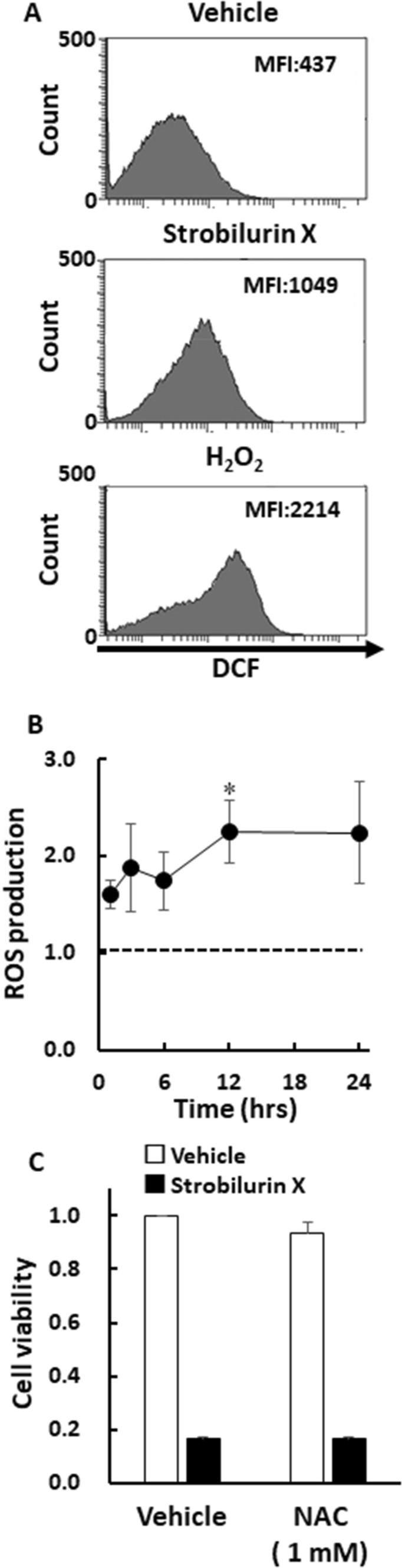
ROS production is not involved in the cytotoxicity induced by strobilurin X. **A** Typical histograms showing the cell numbers with DCF fluorescence after exposure to vehicle (12 h), strobilurin X (6.25 μg/mL, 12 h) and hydrogen peroxide (H_2_O_2_, 0.5 mM, 1 h). The mean fluorescence intensity (MFI) was calculated using the BD FACSDiva^™^ software. **B** The time course of ROS production, indicated as the ratio of each MFI to that of the vehicle at each time point after exposure to strobilurin X. Symbols with vertical lines show the mean ± SEM in three separate experiments. The dashed line shows a baseline equal to the level in the vehicle. *p < 0.05 vs. the vehicle using Student’s *t*-test. **C** The effect of *N*-acetyl cysteine (NAC; 1 mM) on strobilurin X-induced cytotoxicity in A549 cells at 48 h. Cell viability is indicated as the ratio of each value to that of the vehicle without NAC (open columns, vehicle; closed columns, strobilurin X [6.25 μg/mL]). Cells were pretreated with NAC 30 min before exposure to strobilurin X. Columns with vertical lines show the mean ± SEM in three separate experiments

Various anticancer compounds have generated intracellular ROS, which induced cellular damage [23, 24]. The number of A549 cells emitting stronger fluorescence for the ROS indicator dichlorodihydrofluorescein (DCF) increased to the maximum level after 12 h of exposure to strobilurin X, although the mean fluorescence intensity was lower than that of number of hydrogen peroxide (0.5 mM) for 1 h (Fig. 5A and B). The strobilurin X-induced high fluorescent intensity levels were maintained up to 24 h (Fig. 5B). However, *N*-acetyl cysteine, a potent ROS scavenger, failed to attenuate the strobilurin X-induced cytotoxicity in A549 cells (Fig. 5C). These results suggest that strobilurin X induces the production of intracellular ROS, but its effect is not essential for the cytotoxicity.

**Fig. 6:**
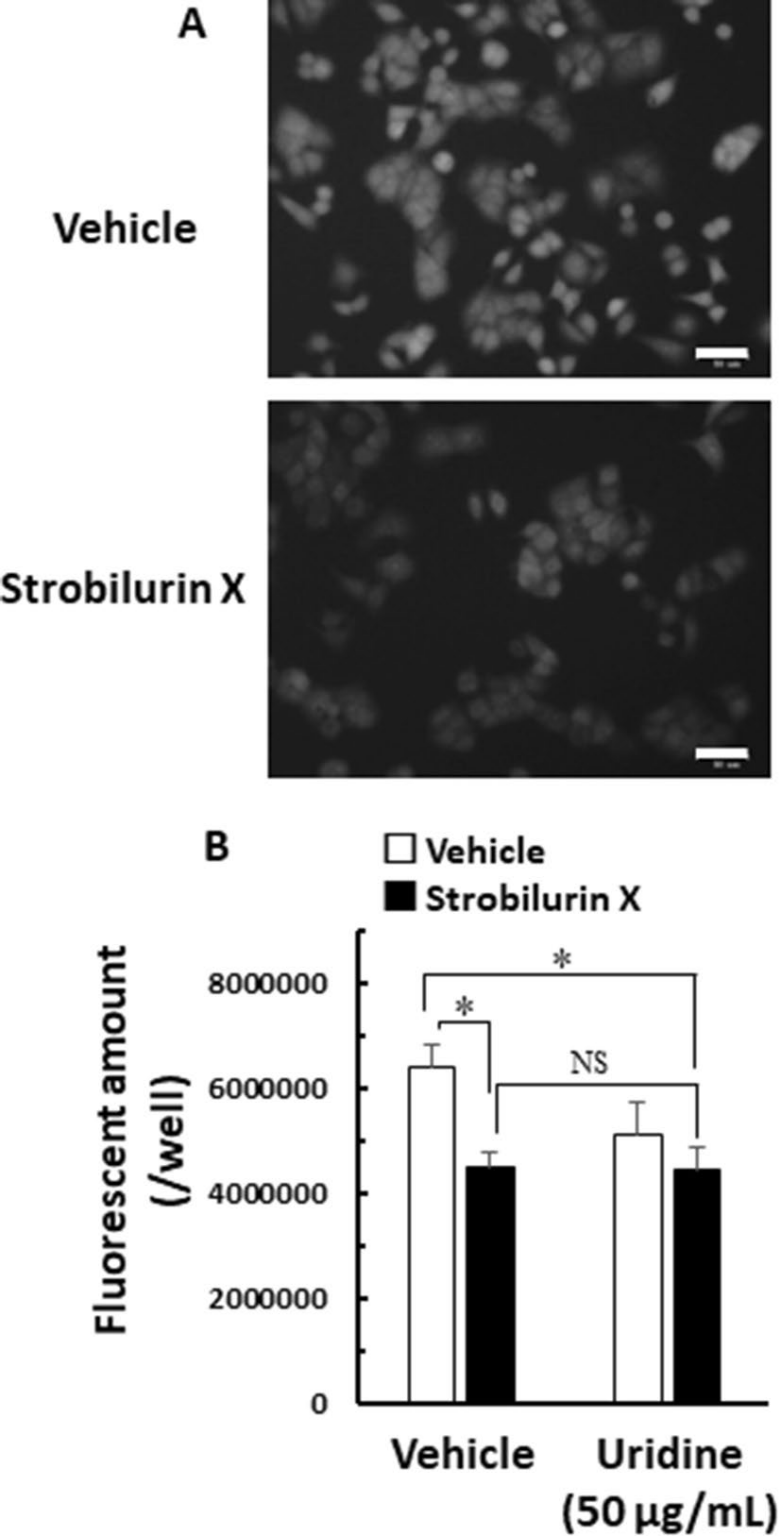
Effect of strobilurin X on protein synthesis in A549 cells. **A** Typical fluorescent microscope images of the protein synthesis activity 2 h after exposure to strobilurin X (upper, vehicle; lower, 25 μg/mL of strobilurin X). White scale bars: 50 μm. **B** The fluorescent amount in each well without (vehicle) or with uridine (50 μg/mL) (open columns, vehicle; closed columns, 25 μg/mL of strobilurin X). Columns with vertical lines show the mean ± SEM in three separate experiments. *p < 0.05 vs. the vehicle using the Games-Hawel test. *NS* not significant

Recently, the link between protein synthesis inhibition and anticancer activity has been focused on certain compounds [25]. Protein synthesis is indispensable in cell proliferation; hence, we evaluated the protein synthesis activity using OPP, which was labeled on the C-terminus of nascent polypeptide chains [26]. Strobilurin X decreased the cell fluorescent intensity labeled by protein synthesis for 2 h in A549 cells, indicating that strobilurin X inhibits protein synthesis (Fig. 6A). Uridine failed to block the inhibition of protein synthesis by strobilurin X (Fig. 6B). These results suggest that strobilurin X can inhibit protein synthesis and mitochondrial respiratory chain complex III.

## Discussion

In this study, we found that the cytotoxic effects of strobilurin X (isolated from *M. venosolamellata)* on cancer cells were greater than those on normal fibroblasts. Moreover, strobilurin X inhibited protein synthesis and mitochondrial respiratory chain complex III in the lung cancer cell line.

The mitochondrial respiratory chain is essential for generating the intracellular concentration of ATP. It is considered as a target for anticancer effects owing to its ability to regulate the metabolism and proliferation of cancer cells [27]. The oxidation of dihydroorotate dehydrogenase by complex III produces orotates, which lead to pyrimidine biosynthesis [28]. The depletion of complex III inhibits de novo pyrimidine synthesis and induces cytotoxicity in cancer cells [15]. A commercially available strobilurin pesticide, azoxystrobin, was reported to induce cytotoxicity, which was entirely rescued by uridine in myelogenous leukemia HL-60RG cells [29]. In the current study, strobilurin X-induced cytotoxicity was partially abolished by uridine, whereas methyl pyruvate did not have any effect in attenuating the strobilurin X-induced cytotoxicity (Fig. 3). Therefore, we hypothesized that other mechanisms, apart from mitochondrial respiratory complex III inhibition, were involved in the cytotoxicity of A549 cells.

Strobilurin X was found to directly suppress protein synthesis in this study (Fig. 6). Strobilurins A and B were reported to attenuate the incorporation of radioactive leucine in Ehrlich carcinoma ascites cells [3]. Direct evidence for the inhibition of protein synthesis by strobilurin X was seen for the first time in the present study. The chemical structure of strobilurin X is different from that of strobilurin A in terms of the presence of a methoxy group at the para position of its benzene ring; it is different from that of strobilurin B at the position of the methoxy group and the Cl substituents and significantly different from that of azoxystrobin [1, 3, 11]. Therefore, the inhibition of protein synthesis may be dependent on the structure of the strobilurins. Nonetheless, future studies identifying the molecular targets and clarifying the structure–activity relationship in protein synthesis are warranted.

Strobilurins are generally used as pesticides but are currently being evaluated as anticancer drugs [30]. Interestingly, azoxystrobin induced cytotoxicity by inhibiting the phosphorylation of PI3K/Akt and Erk [31]. However, the inhibitors of these molecules did not affect the cytotoxic action of strobilurin X in the present study. The cytotoxic activity of strobilurin X may not be related to these cell growth signals in A549 cells.

Natural compounds such as phytochemicals often generate ROS, which evokes cytotoxicity [23, 24]. Strobilurin X induced ROS generation in the current study. It may induce the generation of ROS in mitochondria due to the inhibition of mitochondrial respiratory chain complex III [32]. However, the potent antioxidant *N*-acetyl cysteine failed to suppress the strobilurin X-induced cytotoxicity in this study (Fig. 5). Similarly, mito-TEMPO, an intra-mitochondria ROS scavenger, also failed to block the strobilurin X-induced cytotoxicity (data not shown). Furthermore, strobilurin X did not induce the generation of ROS in HeLa cells despite its cytotoxicity (Fig. 1 and data not shown). Therefore, the generation of ROS by strobilurin X may not be essential for its cytotoxic action. Alternatively, the amount of ROS generated by strobilurin X may not be sufficient to kill A549 cells.

In summary, the inhibition of mitochondrial respiratory chain complex III and protein synthesis was found to be related to the cytotoxic effects of strobilurin X in A549 cells. Strobilurin-related compounds are widely used as pesticides; thus, the drug repositioning may be expected. The findings of this study indicate that strobilurin X may be considered for the development of novel anticancer drugs.

## Data Availability

All data included in this study are available from the corresponding author upon appropriate request.

## Abbreviations

DCF: Dichlorodihydrofluorescein
DMSO: Dimethylsulfoxide
Erk: Extracellular signal-regulated kinase
FAM: 5-Carboxyfluorescein
H_2_DCFDA: 2ʹ,7ʹ—Dichlorodihydrofluorescein diacetate
MAPK: Modulate mitogen-activated protein kinase
MEK: Mitogen activated protein kinase/extracellular signal-regulated kinase kinase
NAC: *N*-Acetyl cysteine
OPP: *O*-Propagyl-puromycin
PBS: Phosphate-buffered saline
PI3K: Phosphoinositide 3-kinase
ROS: Reactive oxygen species
SEM: Standard error of the mean
